# Optimized Organic Fertilization Mitigates Antibiotic Resistance Gene Dissemination in Manure-Amended Soils: A Field Study on Nutrient–Microbiome–Antibiotic Resistance Gene Nexus During Cabbage Reproductive Cycle

**DOI:** 10.3390/antibiotics15090821

**Published:** 2026-08-24

**Authors:** Han Wang, Keqiang Zhang, Muheng Liu, Shenwei Cheng, Cheryl Marie Cordeiro, Erik Sindhøj, Junfeng Liang, Yuanfang Zeng, Shizhou Shen, Suli Zhi

**Affiliations:** 1 Agro-Environmental Protection Institute, Ministry of Agriculture and Rural Affairs, Tianjin 300191, China; 2 National Field Observation and Research Station (Yunnan Dali) for Agro-Ecosystem, Dali 671004, China; 3 Key Laboratory of Low-Carbon Green Agriculture in North China, Ministry of Agriculture and Rural Affairs, Beijing 100193, China; 4 Chifeng Municipal Agriculture and Animal Husbandry Bureau Plant Protection and Plant Quarantine Center, Chifeng 024000, China; 5 University of Chinese Academy of Sciences, Beijing 100049, China; 6 NIBIO, Norwegian Institute of Bioeconomy Research, N-1430 Ås, Norway

**Keywords:** mobile genetic elements, fertilization strategies, risk assessment, horizontal gene transfer, contamination factor

## Abstract

**Background**: Manure-amended agricultural soil is a critical reservoir of antibiotic resistance genes (ARGs), posing escalating threats to environmental health and food safety. However, the temporal trajectories of ARG prevalence throughout the complete reproductive cycle of cash crops, and their mechanistic linkages with fertilization regimes and microbial community succession, remain inadequately understood. **Methods**: To bridge this knowledge gap, we conducted an in situ field experiment over the entire growth period of Chinese cabbage at a long-term manure-amended farm in Tianjin, China. Six contrasting fertilization strategies were evaluated: unfertilized control (CK1), unfertilized baseline control (CK2), traditional full-rate combined manure–chemical fertilization (TF), traditional half-rate combined manure–chemical fertilization (T1), half-dose sole manure fertilizer (T2), and half-dose sole chemical fertilizer only (T3). **Results**: Our results demonstrated that ARG abundance and associated mobile genetic elements (MGEs) exhibited a pronounced transient surge immediately post-fertilization, yet reverted to baseline levels by harvest, revealing a tangible resilience of the soil resistome. Notably, the optimized half-organic fertilization (T2) effectively curtailed the proliferation of manure-derived pathogenic taxa while preserving beneficial keystone phyla (e.g., *Acidobacteria* and *Proteobacteria*), indicating a trade-off between nutrient provisioning and ecological filtering. Co-occurrence network analysis further identified *MB-A2-108*, *Saccharimonadales*, and *Rokubacteriales* as pivotal hosts for multidrug-resistant ARGs, underscoring that microbial interspecific interactions—rather than taxonomic richness alone—are the primary drivers of resistome succession. Quantitative risk assessment confirmed that the T2 regimen reduced the composite ARG contamination index (CF_zone_) by 25% relative to conventional full fertilization (TF), while maintaining comparable cabbage yields. **Conclusions**: Collectively, our findings advocate for precision organic fertilization as a nature-based solution that synchronizes nutrient supply with crop demand, curtails ARG propagation, and mitigates long-term agroecological risks.

## 1. Introduction

The widespread presence and spread of antibiotic resistance genes (ARGs) in agricultural soils have emerged as a pressing global concern, posing critical threats to both ecosystem integrity and human health [1,2]. The integration of crop cultivation with livestock farming has long been advocated as a sustainable strategy for managing livestock waste and improving soil fertility through nutrient recycling [3]. However, this practice harbors an inherent paradox: while organic fertilization enhances soil productivity, the extensive use of veterinary antibiotics in animal husbandry introduces both antibiotic residues and ARG-harboring microorganisms into arable lands, inadvertently transforming agricultural soils into vast reservoirs of resistance determinants [4]. The misuse and overuse of antibiotics not only accelerate bacterial resistance but also facilitate the environmental dissemination of ARGs through manure-based fertilization, creating potential entry points into the food chain via contaminated crops and water resources [5].

Beyond soil, ARGs have been detected in surface water and groundwater due to runoff from manure-applied fields, posing risks to drinking water quality and aquatic ecosystems [6]. Antibiotic residues in irrigation water can be absorbed by plants, leading to potential human exposure through agricultural produce. Studies have shown that ARGs persist in leafy vegetables, raising concerns about their role in antimicrobial resistance in clinical settings [7]. Additionally, ARGs may enter the human microbiome via ingestion of contaminated food, water exposure, or soil contact, further complicating public health challenges. Understanding these pathways is critical for developing mitigation strategies that balance agricultural productivity with environmental and human health protection.

With the global adoption of precision agriculture and sustainable soil management frameworks, research has increasingly prioritized the efficient utilization of organic fertilizers to enhance soil fertility while minimizing associated environmental risks. Large-scale livestock operations commonly rely on manure application as a cost-effective means of nutrient recycling; however, excessive fertilization remains pervasive in vegetable production systems, driven by high crop nitrogen demands and the slow-release characteristics of organic amendments [8]. Overapplication of organic fertilizers has been unequivocally linked to elevated antibiotic residues and increased abundance of resistant bacteria in soil ecosystems [9,10]. A study by Zhang et al. detected 49 distinct antibiotics in farmland soils receiving organic amendments [11], while Marti et al. and Wu et al. reported continuous organic amendment application can inadvertently introduce trace metals such as copper (Cu) and zinc (Zn) into agricultural soils [12,13]. Heavy metals exert strong co-selective pressure on microbial communities [14]. Mechanistically, sublethal heavy metal concentrations stimulate intracellular reactive oxygen species (ROS) generation, increase cell membrane permeability, and upregulate SOS response pathways, thereby facilitating the horizontal gene transfer (HGT) of ARGs via mobile genetic elements (MGEs) such as plasmids, integrons, and transposons [15]. Consequently, establishing optimal fertilization thresholds that balance nutrient provisioning with the mitigation of ARG dissemination has become a central challenge for sustainable agricultural management.

While the immediate effects of organic fertilizers on soil microbial diversity and composition have been relatively well documented, the long-term dynamics of ARG persistence and transmission under contrasting fertilization regimes remain incompletely resolved. The introduction of organic fertilizers may alter selective pressures and ecological niches within soil, either promoting or attenuating ARG transmission depending on nutrient availability, microbial competition, and community assembly processes. However, the majority of existing studies have relied on single time-point samplings or short-term microcosm experiments, providing static snapshots that fail to capture the dynamic succession of ARGs throughout complete crop reproductive cycles [16]. This temporal dimension is critically important, as plant growth stages impose fluctuating selective pressures through rhizodeposition, nutrient depletion, and shifts in microbial community structure, all of which may profoundly influence ARG abundance and mobility.

This study investigated the effects of different fertilizer application patterns on ARG abundance, soil nutrient dynamics, and microbial community structure in a Chinese cabbage production system. A field trial was conducted on a long-term manure-applied farm in Tianjin, China, with a specific focus on the half-organic fertilizer (T2) treatment to assess its potential for reducing ARG risks while maintaining soil fertility. The study aimed to elucidate the temporal variations in ARG abundance during the cabbage reproductive period, evaluate microbial community shifts under different fertilization strategies, and identify key microbial hosts associated with ARG transmission. The findings provide insights into the spatial and temporal evolution of the nutrient–microbe–ARG system under manure application, contributing to precision fertilization practices that optimize sustainability while mitigating antimicrobial resistance spread.

## 2. Materials and Methods

### 2.1. Experimental Site

The field experiment was conducted at a long-term manure-amended farm located in Jinghai District, Tianjin, China (Basic soil physicochemical properties are shown in Table S1), a region renowned for intensive vegetable cultivation and heavy reliance on organic fertilization. This site was selected due to its extended history of manure application, rendering it ideally suited for investigating the transmission dynamics of antibiotic resistance genes (ARGs) in agricultural soils. The experiment was carried out in 2022 and 2023, from July to November each year, encompassing the complete reproductive cycle of Chinese cabbage.

The farm operates a closed-loop organic fertilization system, wherein solid manure undergoes static composting prior to field application, while the liquid fraction is separated from raw manure and subjected to storage and fermentation in manure lagoons. This integrated management practice aligns with sustainable agricultural principles, enabling direct nutrient recycling through manure return and facilitating the coupling of crop production with livestock operations.

### 2.2. Fertilization Design

The experimental site followed a baseline fertilization regime, applied in spring in accordance with local farming practices. To assess the impact of fertilization strategies on ARGs, six treatment groups were established: CK1 (Control Check 1): No fertilizer application; CK2 (Control Check 2): Basal fertilizer only, no additional fertilizer; TF (Traditional Fertilization): Full-amount organic and chemical fertilizer mix; T1: Half-amount organic and chemical fertilizer mix; T2: Half-amount organic fertilizer only; T3: Half-amount chemical fertilizer only. T2 was selected as a key experimental focus because it represents a realistic mitigation strategy for reducing ARG contamination while maintaining soil fertility [17]. Many agricultural policies, including those in the EU and China, encourage reduced manure inputs to prevent excessive nitrogen loading and groundwater contamination. Investigating T2 provides insight into how lower manure application rates can balance productivity with environmental risk mitigation. Nutrient inputs for each treatment are detailed in Table 1. Each treatment was randomly assigned in triplicate to minimize bias. Plot dimensions were 7.02 m^2^ (3.9 m × 1.8 m), with buffer zones of equal area established between adjacent plots. Chinese cabbage (AoLv 75), a widely cultivated variety in the region, was used to ensure reproducibility of results. The basal fertilizer was applied according to local farmers’ conventional fertilization rates and incorporated into the 0–20 cm topsoil before sowing. Chemical fertilizers were supplemented as needed, using calcium superphosphate (Ca(H_2_PO_4_)_2_·H_2_O and CaSO_4_, 12% P_2_O_5_) and potassium sulfate (K_2_SO_4_, 51% K_2_O).

### 2.3. Sample Collection and Processing

Soil and plant samples were collected at four discrete phenological stages—pre-planting, heading, rosette, and harvesting—representing the key growth phases of Chinese cabbage. For soil analysis, a five-point composite sampling strategy was employed within each plot to collect samples from the 0–20 cm topsoil layer; the composite samples were then air-dried, sieved through 1 mm and 0.15 mm mesh screens, and stored in a cool dark place indoors for routine physicochemical analysis, while subsamples designated for molecular analysis were immediately frozen at −80 °C until DNA extraction. For plant analysis, Chinese cabbage samples were thoroughly chopped, homogenized, and divided into subsamples following the quartering method; one subsample was oven-dried at 65 °C, ground, and sieved through a 0.25 mm mesh for nutrient determination, whereas another subsample was rinsed, immediately frozen at −80 °C, and preserved for subsequent molecular analyses.

### 2.4. Determination of Chemical Properties

Soil pH was measured in a 1:5 (*w*/*v*) soil-to-deionized water suspension using a calibrated pH meter (Model FE20, Mettler-Toledo Instruments (Shanghai) Co., Ltd., Shanghai, China) [18]. Total nitrogen (TN) was determined by the Kjeldahl digestion method using an automatic Kjeldahl apparatus (Model UDK159, Beijing Yingsheng Hengtai Technology Co., Ltd., Beijing, China) [19]. Ammonium nitrogen (NH_4_^+^-N) and nitrate nitrogen (NO_3_^−^-N) were extracted with 2 M KCl and quantified using a flow injection analyzer (FIA-6000+; Beijing Jitian Instrument Co., Ltd., Beijing, China). Available phosphorus (AP) was determined by the Olsen method using a spectrophotometer (DR6000; Hach Company, Loveland, CO, USA) [20]. Soil heavy metals (Zn, Cu, Pb, Cr, Cd, As) were digested using an HNO_3_-HClO_4_-HF mixture and quantified via inductively coupled plasma mass spectrometry (ICP-MS, Agilent 7500ce, Agilent Technologies, Santa Clara, CA, USA).

Sixteen target antibiotics across four major classes (tetracyclines, sulfonamides, fluoroquinolones, macrolides) were extracted from 2.0 g freeze-dried soil or plant tissue using ultrasonic-assisted extraction with an acetonitrile-phosphate buffer solution (*v*/*v* = 1:1). Extracts were purified via HLB solid-phase extraction (SPE) cartridges (Waters, Milford, MA, USA), eluted with methanol, concentrated under a mild N_2_ stream, and re-dissolved in 1.0 mL methanol. Target concentrations were quantified using ultra-performance liquid chromatography-tandem mass spectrometry (UPLC-MS/MS, Agilent 1290/6460, Agilent Technologies, Santa Clara, CA, USA) equipped with an electrospray ionization (ESI) source operating in positive mode. Matrix-matched standard curves were used to ensure recovery rates between 82% and 108%.

### 2.5. Real-Time qPCR Assay

DNA was extracted from soil and plant samples using the Soil Genomic DNA Extraction Kit (DcP336, Tengen Biochemical Technology, Beijing, China). The study targeted six ARG classes and three mobile genetic elements (MGEs), as shown in Table 2. MGEs included *intI*1, *Tn*916. and *ISCR*1, whose primers for all genes are listed in Table S2. Amplification reactions (20 μL) contained 10 μL PowerUp SYBR Green Master Mix, 0.4 μL each of forward and reverse primers (10 μM), 1.0 μL DNA template, and 8.2 μL nuclease-free water. Thermal cycling conditions consisted of 95 °C for 10 min, followed by 40 cycles of 95 °C for 15 s and 60 °C for 1 min. Standard curves were generated using ten-fold serial dilutions of plasmid vectors containing target gene fragments (R^2^ > 0.99, efficiency = 92–105%). Absolute gene copy numbers were normalized to dry soil weight (copies g^−1^).

### 2.6. MiSeq Sequencing

Bacterial community composition was evaluated by targeting the V3–V4 hypervariable regions of the 16S rRNA gene using primers 338F (5′-ACTCCTACGGGGAGGCAGCAG-3′) and 806R (5′-GGACTACNNGGGGTATCTAAT-3′). Purified amplicons were pooled in equimolar concentrations and paired-end sequenced (2 × 300 bp) on an Illumina MiSeq platform (sequenced by Beijing Ovison Gene Technology Co., Ltd., Beijing, China). Raw fastq files were quality-filtered using FastQC (Babraham Institute, Cambridge, UK) and processed via QIIME (Version 1.8.0). Sequences were clustered into Operational Taxonomic Units (OTUs) at 97% sequence similarity using UPARSE (USEARCH v11, Drive5 Software, Berkeley, CA, USA), and taxonomic classification was performed against the SILVA v138 database [21,22,23].

### 2.7. Statistical Analysis, Network Analysis, and Risk Assessment

Data were analyzed using two-way analysis of variance (ANOVA) to assess the main effects of fertilization treatment, sampling stage, and their interaction. Tukey’s Honest Significant Difference (HSD) test was used for post hoc mean comparisons (*p* < 0.05). Co-occurrence patterns among ARGs, MGEs, and microbial genera were evaluated using Spearman’s rank correlations. To reduce network complexity, taxa with relative abundances < 0.01% were excluded. Robust correlations with |*p*| > 0.70 and *p* < 0.01 (Benjamini–Hochberg false discovery rate adjusted) were incorporated into network construction. Visualizations and topology metrics were generated in Gephi (v0.9.2).

Environmental risk from ARGs was quantified using the Contamination Factor (CF_i_) and composite Contamination Index (CF_zone_):

$${\mathrm{CF}}_{i}\;=\;\frac{C_{i}}{C_{i}\;-\;backgound}$$

$${\mathrm{CF}}_{\mathrm{zone}}=\frac{\Sigma {CF}_{i}}{n}$$

where C*_i_* represents the absolute abundance of ARG*_i_* at a given stage, $C_{i}\;-\;backgound$ is the baseline ARG abundance prior to fertilization, and n is the total number of evaluated ARGs (n = 16). CF_zone_ ≤ 1.0 indicates negligible contamination, 1.0 < CF_zone_ ≤ 2.0 represents moderate contamination, and CF_zone_ > 2.0 signifies severe contamination.

Ecotoxicological Risk Quotients (RQ) for individual antibiotics in soil and crop tissues were calculated as (Tables S3 and S4):

$${\mathrm{RQ}}_{\mathrm{soil}}\;=\;\frac{{MEC}_{soil}\;(mg/kg)}{{PNEC}_{soil}\;(mg/kg)}$$

where MEC is the measured environmental concentration, and PNEC is the predicted no-effect concentration derived from ecotoxicology databases. Total risk (ΣRQ) was calculated as the sum of individual antibiotic RQ values. RQ < 0.1, 0.1 ≤ RQ < 1.0, and RQ ≥ 1.0 represent low, moderate, and high risk, respectively.

## 3. Results and Discussions

### 3.1. Effect of Different Fertilization Treatments on Soil Nutrient Dynamics

Soil nutrient availability influences plant growth, microbial composition, and nitrogen cycling in agricultural systems. This section examines how fertilization treatments affected total nitrogen (TN), nitrate nitrogen (NO_3_^−^-N), ammonium nitrogen (NH_4_^+^-N), available phosphorus (AP), and soil pH during the reproductive period of Chinese cabbage.

#### 3.1.1. Temporal Changes in Soil Nitrogen Content

**Figure 1:**
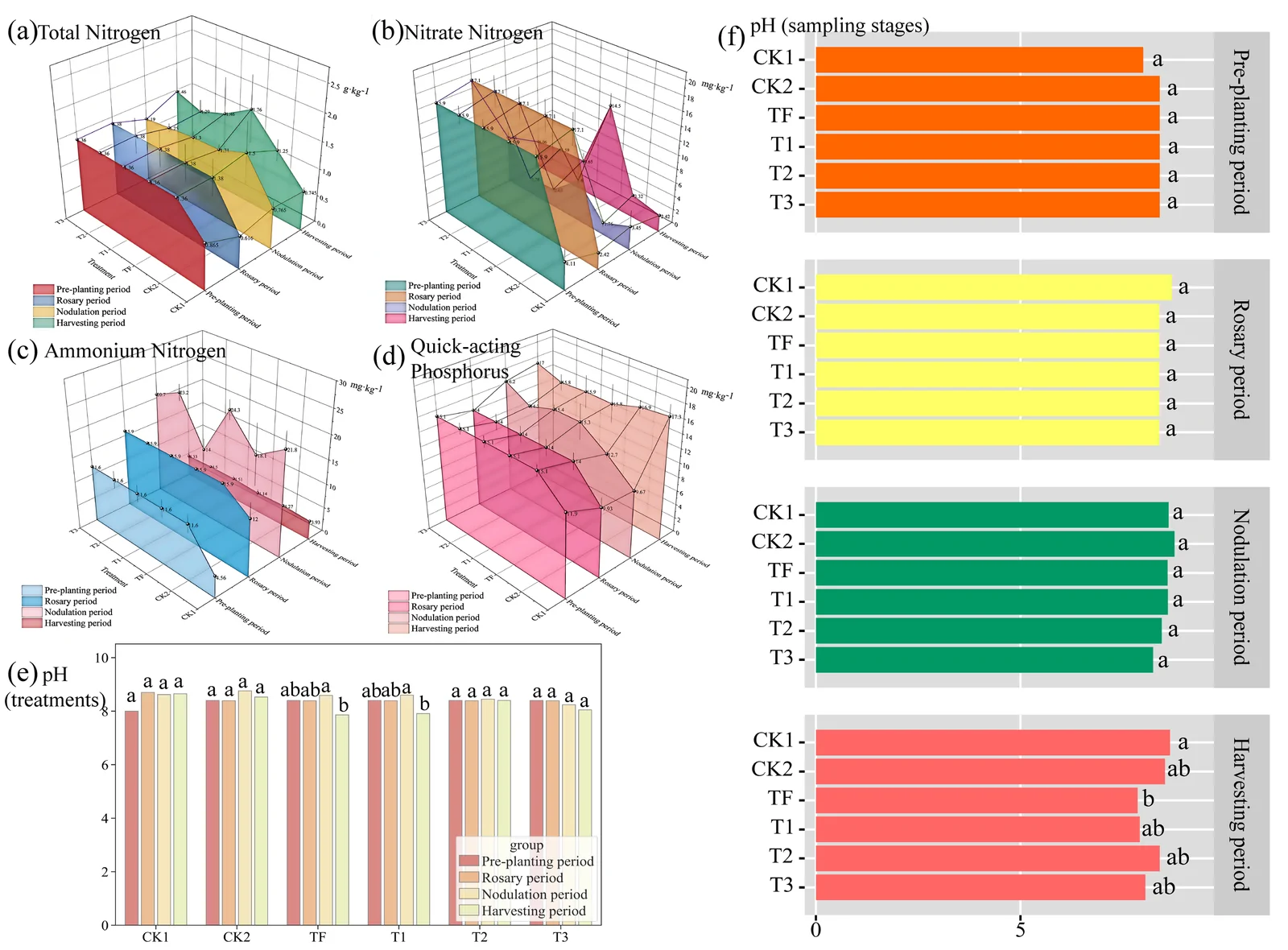
Effects of different fertilization patterns on soil total nitrogen (**a**), nitrate nitrogen (**b**), ammonium nitrogen (**c**), and quick-acting phosphorus (**d**); effects of fertilization treatments (**e**) and sampling stages (**f**) on soil pH. Note: Figure (**e**) compares differences among different reproductive stages under the same treatment conditions, while figure (**f**) compares differences among different treatments within the same reproductive stage.

Fertilization strategies and phenological stages significantly altered soil chemical properties (*p* < 0.05; Figure 1a–d). Soil TN, NO_3_^−^-N, NH_4_^+^-N, and AP increased following basal fertilizer application, reaching peak values during the rosette stage in organic-amended plots. At heading stage, TN levels were similar across TF, T1, T2, and T3, but at harvest, TN followed the order TF > T1 > T3 > T2 > CK2 > CK1, with TF-treated soils containing 2.36 times more TN than CK1 (*p* < 0.01). Pairwise Tukey HSD tests confirmed that total nitrogen (TN) levels within individual treatments did not differ significantly between the heading and harvest stages (*p* > 0.05), whereas available mineral nitrogen NO_3_^−^-N underwent intense temporal depletion due to active crop uptake. This confirms that while TF enhances TN retention, moderate fertilization (T2) maintains adequate TN levels.

NO_3_^−^-N exhibited stage-dependent fluctuations. Basal fertilization increased NO_3_^−^-N from 4.11 mg·kg^−1^ to 15.89 mg·kg^−1^, but concentrations declined during heading stage due to plant uptake. At harvest, TF-treated soils retained the highest NO_3_^−^-N levels (14.5 mg·kg^−1^), while T2 and other reduced fertilization treatments exhibited lower NO_3_^−^-N accumulation (*p* < 0.05). CK1 and CK2 showed minor fluctuations but returned to rosette-stage levels at harvest, indicating that excessive fertilization may contribute to nitrate retention, increasing the risk of leaching.

NH_4_^+^-N increased in fertilized soils during heading stage, by harvest, NH_4_^+^-N levels remained elevated across treatments, including CK1, which peaked at 17.3 mg·kg^−1^, implying that microbial activity and plant uptake regulate ammonium availability over time.

#### 3.1.2. Phosphorus Availability and pH Variations

AP followed distinct temporal patterns. All fertilized treatments, except T1, showed an AP increase during the rosette stage, with TF reaching the highest levels. However, AP concentrations declined significantly at harvest, with no statistically significant differences between treatments, suggesting that phosphorus is rapidly absorbed by plants or immobilized in the soil.

Soil pH exhibited treatment-dependent variations (Figure 1e,f). CK1 maintained the highest pH at the pre-planting and rosette stages. Fertilized treatments showed a slight pH increase at heading stage, followed by a decline at harvest, with the sharpest drop observed in T3 during heading stage and in TF and T1 at harvest. The pH decline in high-fertilization treatments likely results from enhanced nitrification and organic acid production by microbial activity. These findings align with previous studies linking long-term chemical fertilization to soil acidification, negatively impacting microbial communities and nutrient cycling [24].

#### 3.1.3. Insights on Nutrient Regulation in Fertilization Management

Moderate fertilization enhances soil nutrient content without excessive accumulation, while over-fertilization leads to nutrient surpluses that may not be fully utilized by crops. Excess nitrogen in TF-treated soils increases the risk of volatilization as ammonia (NH_3_) or leaching as NO_3_^−^, with potential impacts on greenhouse gas emissions and groundwater contamination [25]. Additionally, long-term nitrogen fertilization alters NH_4_^+^-N retention in the 0–20 cm layer [8], indicating shifts in nitrogen cycling.

The decline in phosphorus availability over time highlights the need for optimized phosphorus management to prevent depletion in later growth stages. Soil pH dynamics further emphasize the importance of balancing organic and inorganic fertilizers to sustain microbial stability and long-term soil fertility.

### 3.2. Changes in Heavy Metal and Antibiotic Content During the Reproductive Period of Chinese Cabbage

#### 3.2.1. Antibiotic Residues in Soil Across Growth Stages

**Figure 2:**
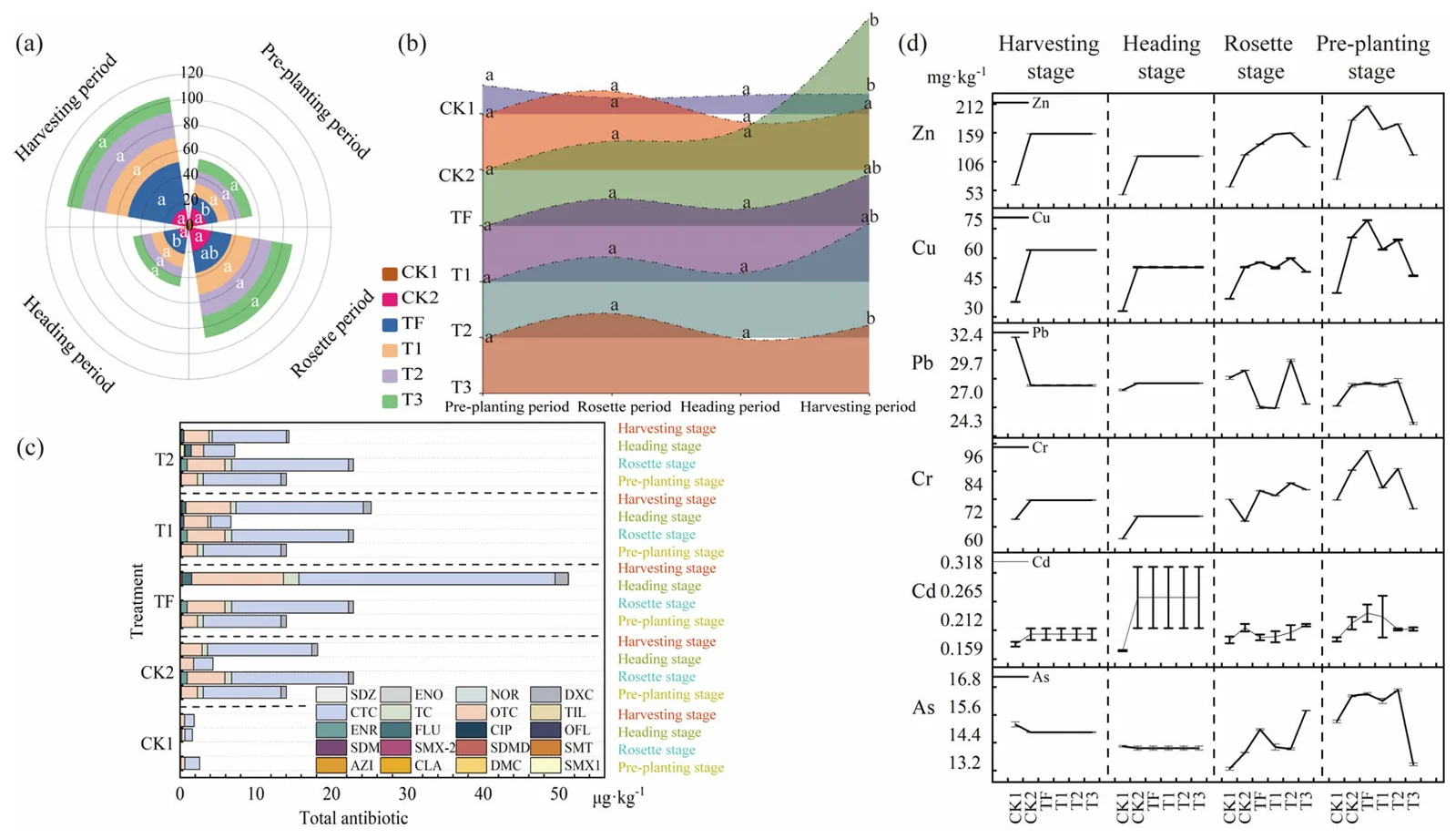
Effects of different fertilization patterns on total antibiotic residues across growth stages (**a**), individual antibiotic residues (**b**), contents of different antibiotic subtypes (**c**), and heavy-metal concentrations (**d**). Note: Figure (**a**) compares differences among different reproductive stages under the same treatment conditions; For panel (**c**), several antibiotic subtypes were below the detection limit in certain samples and were therefore not presented in the figure.

Antibiotic residues varied significantly across treatments and growth stages (Figure 2a,b). TF exhibited the highest total residues, followed by T1, T2, and T3, indicating a direct correlation between organic manure application and antibiotic accumulation. Among reduced-fertilization treatments, T1 retained the most antibiotics, while T3 had the lowest levels, suggesting that lower organic fertilizer inputs reduce antibiotic accumulation.

Residues peaked at harvest, indicating gradual accumulation throughout the crop cycle. TF-treated soils contained the most antibiotic subtypes (six) at harvest (Figure 2c), while CK1 (non-fertilized control) had no detectable antibiotics, confirming manure as the primary source. Tetracyclines, particularly chlortetracycline (CTC) and oxytetracycline (OTC), were the most persistent, aligning with findings that these antibiotics degrade slowly and accumulate over time [26]. The frequent use of tetracyclines in pig farming exacerbates their presence in manure-amended soils. These residues create selective pressure, promoting resistant bacteria and facilitating horizontal ARG transfer within microbial communities [27].

These results highlight the dual impact of organic fertilizers—enhancing soil fertility while introducing persistent antibiotics. Reducing organic fertilizer inputs can minimize antibiotic accumulation and mitigate ARG propagation risks.

#### 3.2.2. Heavy Metal Accumulation Across Growth Stages

Heavy metal concentrations were significantly influenced by fertilization (*p* < 0.01; Figure 2d). Specifically, one-way ANOVA demonstrated that manure application exerted a highly significant effect on soil Cu accumulation (*p* < 0.01) and Cd enrichment (*p* < 0.01), whereas As, Cr, and Pb concentrations showed no statistically significant differences across treatments (*p* > 0.05). Zn, Cu, Cr, and As levels decreased during the rosette stage due to plant uptake, while Pb and Cd concentrations declined slightly in CK treatments but increased in fertilized treatments over time. Zn and Cu showed the highest accumulation, primarily due to their widespread use in livestock feed supplements. Excess Zn and Cu from manure persist in soil, accumulating with repeated applications [28].

Cu and Zn concentrations in CK soils differed by 87.90–105.38% compared to fertilized treatments before and after the rosette stage. By harvest, TF-treated soils had the highest Cu and Zn levels, nearing the risk screening threshold of China’s GB15618-2018 soil pollution standards [29]. Continued accumulation could exceed safe limits, leading to phytotoxicity and reduced soil fertility.

#### 3.2.3. Implications for Sustainable Soil Management

While heavy metal concentrations remained within national safety standards, long-term organic fertilizer use may lead to cumulative enrichment, necessitating careful manure application monitoring [28]. Antibiotic residues, though not immediately hazardous, can persist and exert prolonged selective pressure on microbial communities, fostering ARG proliferation.

Reducing organic fertilizer inputs while maintaining crop productivity is the most effective mitigation strategy. T2, with a 50% reduction in organic fertilization, significantly lowered heavy metal and antibiotic accumulation while sustaining cabbage growth. These findings support a precision fertilization strategy that optimizes nutrient availability while minimizing contamination risks in long-term manure application systems.

### 3.3. Effect of Different Fertilization Treatments on Cabbage

Fertilization plays a key role in crop productivity and nutrient distribution. This section evaluates the effects of different fertilization treatments on cabbage yield and nutrient accumulation, highlighting strategies to maintain productivity while improving nutrient use efficiency.

#### 3.3.1. Effects on Cabbage Yield

Cabbage yield data (Table 3) show that all fertilized treatments (TF, T1, T2, T3) significantly outperformed control groups (CK1, CK2), though no statistical differences were observed among fertilized treatments. TF and T2, which received the highest manure applications, had the greatest yield increases, with yields ranking TF > T2 > T3 > T1. Despite receiving 50% less nitrogen than TF, T1, T2, and T3 maintained comparable yields, indicating that applying half the conventional nitrogen rate (185 kg·hm^−2^) sustains productivity while reducing fertilizer costs and environmental risks.

These findings align with [25], who reported that beyond a certain threshold, additional nitrogen does not enhance productivity. Furthermore, cabbage grown in soils with long-term organic fertilization showed yield increases even when no additional manure was applied during the rosette stage, suggesting that residual soil fertility contributes to nutrient availability, reinforcing the benefits of sustained organic fertilization.

#### 3.3.2. Effects on Nutrient Distribution in Cabbage

**Figure 3:**
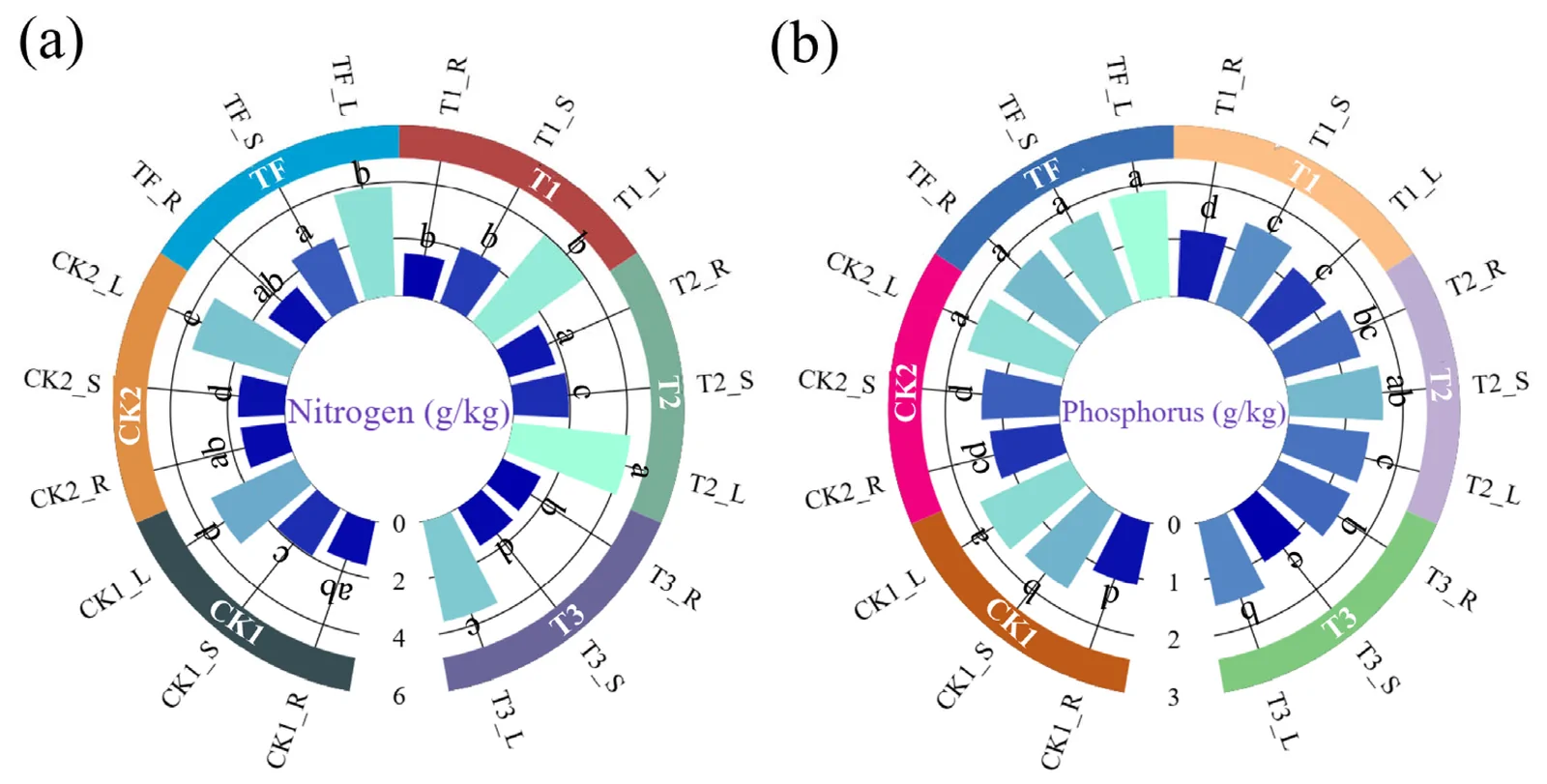
Effect of different fertilization treatments on nitrogen (**a**) and phosphorus (**b**) contents in different parts of cabbage. L, leaf; S, stem; R, root. The suffixes 1, 2 and 3 following each treatment denote pre-planting, rosette stage, heading stage, and harvesting stage, respectively. Note: Figures (**a**) and (**b**) compare the differences observed in different plant tissue parts under different treatments, respectively.

Nitrogen and phosphorus distribution within different plant organs (leaf, stem, root) is illustrated in Figure 3. Nutrient accumulation followed the pattern: leaf > stem > root, with the highest uptake in the edible parts of the plant.

For nitrogen content (Figure 3a), leaf nitrogen was significantly higher than in the stem and root across all treatments (*p* < 0.01). Fertilized treatments increased leaf nitrogen levels, ranking T2 > T1 > TF > T3 > CK2 > CK1. Leaf nitrogen under T2 was significantly higher than under CK1, CK2, and T3 (*p* < 0.01). In stems, nitrogen followed TF > T1 > T2 > CK1 > CK2 > T3, while in roots, levels were mostly stable except for higher values in T2 and lower in T1.

For phosphorus content (Figure 3b), phosphorus accumulation was highest in leaves, followed by stems and roots. TF-treated plants had significantly higher phosphorus content across all organs compared to other treatments (*p* < 0.01). In CK1 and CK2, phosphorus was concentrated in leaves, whereas fertilized treatments showed more balanced distribution across plant tissues.

These results confirm that fertilization enhances nutrient uptake, particularly in edible parts of cabbage. However, TF had the highest nitrogen accumulation but also required the greatest fertilizer input, raising concerns about nutrient use efficiency and environmental impact. T2, which received half the organic fertilizer input of TF, achieved comparable nitrogen uptake, demonstrating that reducing fertilizer inputs does not compromise nutrient utilization efficiency. This finding has economic and environmental significance, as T2 minimizes fertilizer costs while reducing nutrient runoff risks.

### 3.4. Effect of Different Fertilization Patterns on the Abundance of ARGs During the Reproductive Period of Chinese Cabbage

The spread of antibiotic resistance genes (ARGs) in agricultural soils poses risks to soil health and ecological stability. This section examines how different fertilization strategies influence the abundance of ARGs and mobile genetic elements (MGEs) over the reproductive cycle of Chinese cabbage, with a focus on manure application and horizontal gene transfer (HGT) among microbial communities.

#### 3.4.1. Absolute Abundance of ARGs in Soil

**Figure 4:**
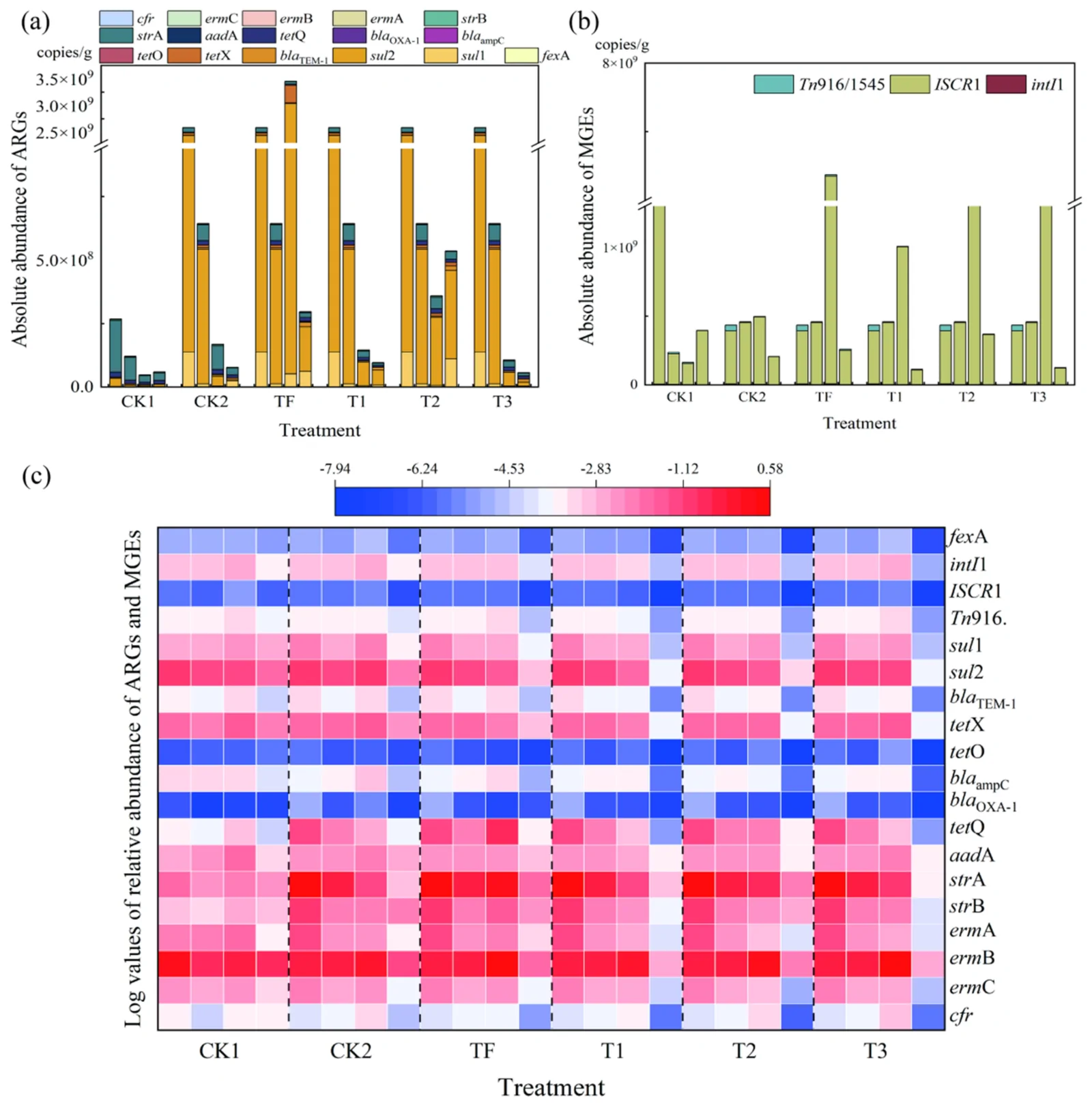
Absolute abundance of ARGs (**a**) and MGEs (**b**) over the reproductive period under different fertilization treatments; relative abundance of ARGs and MGEs (**c**). Samples in each treatment (left to right): pre-planting, rosette, heading, harvesting. Note: Two-way ANOVA indicated that fertilization treatment significantly affected the absolute abundances of *sul*1, *sul*2 (*p* < 0.01), *tet*X, and *tet*O (*p* < 0.01), whereas treatment differences for MGEs (*intI*1, *ISCR*1, *Tn*916/1545) were not statistically significant (*p* > 0.05). Growth stage had a highly significant effect on both ARGs and MGEs (*p* < 0.01). Note: In some samples, the detection levels for several resistance genes were below the limit of detection and are therefore not shown in the figure (**a**,**b**).

Figure 4a shows that reduced organic fertilizer treatments (T1, T2, T3) significantly lowered ARG abundance during heading stage, with T3 exhibiting the lowest levels. This suggests that reducing manure inputs mitigates ARG accumulation.

Among ARG subtypes, *sul*2 was the most enriched before fertilization but declined over time, indicating that while manure contributes to ARG enrichment, microbial competition gradually suppresses some resistance genes. A short-term spike in *sul*2 abundance after fertilization suggests initial manure-derived ARG proliferation, followed by microbial suppression at harvest.

The absolute abundance of *str*A in CK before planting was 2.04 × 10^8^ copies/g, significantly higher than in other treatments, indicating that ARGs persist in soil even without fertilizer application, likely due to previous exposure to antibiotic residues or ARG-harboring microbial populations.

#### 3.4.2. Enrichment of MGEs and Horizontal Gene Transfer Potential

Figure 4b highlights MGEs’ role in facilitating ARG transfer. *ISCR*1 was the most abundant MGE (10^8^–10^9^ copies/g), confirming its dominant role in ARG mobility.

During the rosette stage, reduced nitrogen application (T1, T2, T3) significantly lowered MGE enrichment, supporting the hypothesis that excessive fertilization exacerbates ARG spread by increasing microbial interactions. T1 was particularly effective in reducing MGEs, suggesting that balanced nutrient inputs curb ARG transmission via HGT.

During heading stage, MGE abundance surged across all fertilized treatments before declining at harvest, mirroring increased microbial activity and nutrient availability. The subsequent decline at harvest suggests that native microbial communities outcompete manure-derived bacteria, reducing ARG transmission over time.

#### 3.4.3. Relative Abundance of ARGs Across Growth Stages

Figure 4c shows ARG fluctuations throughout the reproductive cycle. Sixteen ARGs and three MGEs were consistently detected in surface soils, confirming that year-round manure application sustains ARG reservoirs, even when fertilizer inputs are reduced.

Key patterns observed: ARGs peaked during heading stage, particularly *erm*-ARGs (*erm*A, *erm*B, *erm*C), *sul*-ARGs (*sul*1, *sul*2), and *str*-ARGs (*str*A, *str*B, *aad*A). ARG levels declined at harvest, particularly in T3, indicating microbial competition reduces some ARGs over time.

These results align with [3], showing initial manure-derived ARG proliferation followed by gradual suppression as microbial ecosystems adjust. However, persistent ARGs suggest manure creates a long-term resistance reservoir, sustaining ARG transfer risks.

#### 3.4.4. Role of MGEs in ARG Dissemination

MGEs *ISCR*1 and *intI*1 were persistently detected across all sampling stages, with their abundances peaking at the heading stage concurrently with ARG enrichment. Such consistent temporal variation suggested that shifts in microbial community characteristics during crop growth might be partially correlated with the accumulation of soil ARGs. The input of manure-borne microbes at the rosette stage may contribute to the increased ARG abundance observed at the heading stage. At harvest, indigenous soil microorganisms gradually dominated the community, suppressed manure-derived microbial populations, and consequently reduced the overall ARG abundance [11].

As a key genetic element with the capacity to capture exogenous genes, the persistent presence of class 1 integron *intI*1 indicated a potential background for gene exchange in soil [6,7]. Higher ARG/16S rRNA and *intI*1/16S rRNA ratios corresponded to an elevated potential for genetic interaction [30,31,32,33]. Collectively, different fertilization regimes not only altered the abundance of soil ARGs but also modulated the genetic interaction characteristics of soil microbial communities.

#### 3.4.5. Implications for Sustainable Fertilization and ARG Management

While organic fertilizers enrich soil, they introduce and sustain ARG reservoirs, increasing antimicrobial resistance risks. Although ARG abundance declined over time, repeated manure applications contribute to cumulative resistance spread.

T3 (half chemical fertilizer) had the lowest ARG abundance at harvest, indicating that reducing organic inputs curbs ARG persistence. T1 (half organic and chemical mix) significantly reduced MGE enrichment, highlighting balanced nutrient applications limit ARG mobility. TF (full fertilization) led to the highest ARG and MGE accumulation, confirming that excessive manure use amplifies ARG risks.

These insights underscore the need for precision fertilization techniques to balance soil fertility, crop yield, and microbial health while minimizing ARG spread.

### 3.5. Effect of Different Fertilization Patterns on the Soil Microbial Community

Soil microbial communities play a central role in nutrient cycling, soil health, and the spread of antibiotic resistance genes (ARGs). Fertilization influences microbial composition by altering soil nutrient availability, pH, and microbial competition. This section evaluates the impact of different fertilization treatments on microbial community abundance, diversity, and potential hosts for ARGs, complementing the previous findings on soil nutrient changes, heavy metal accumulation, and ARG transmission.

#### 3.5.1. Effect of Fertilization Patterns on Microbial Community Abundance and Composition

**Figure 5:**
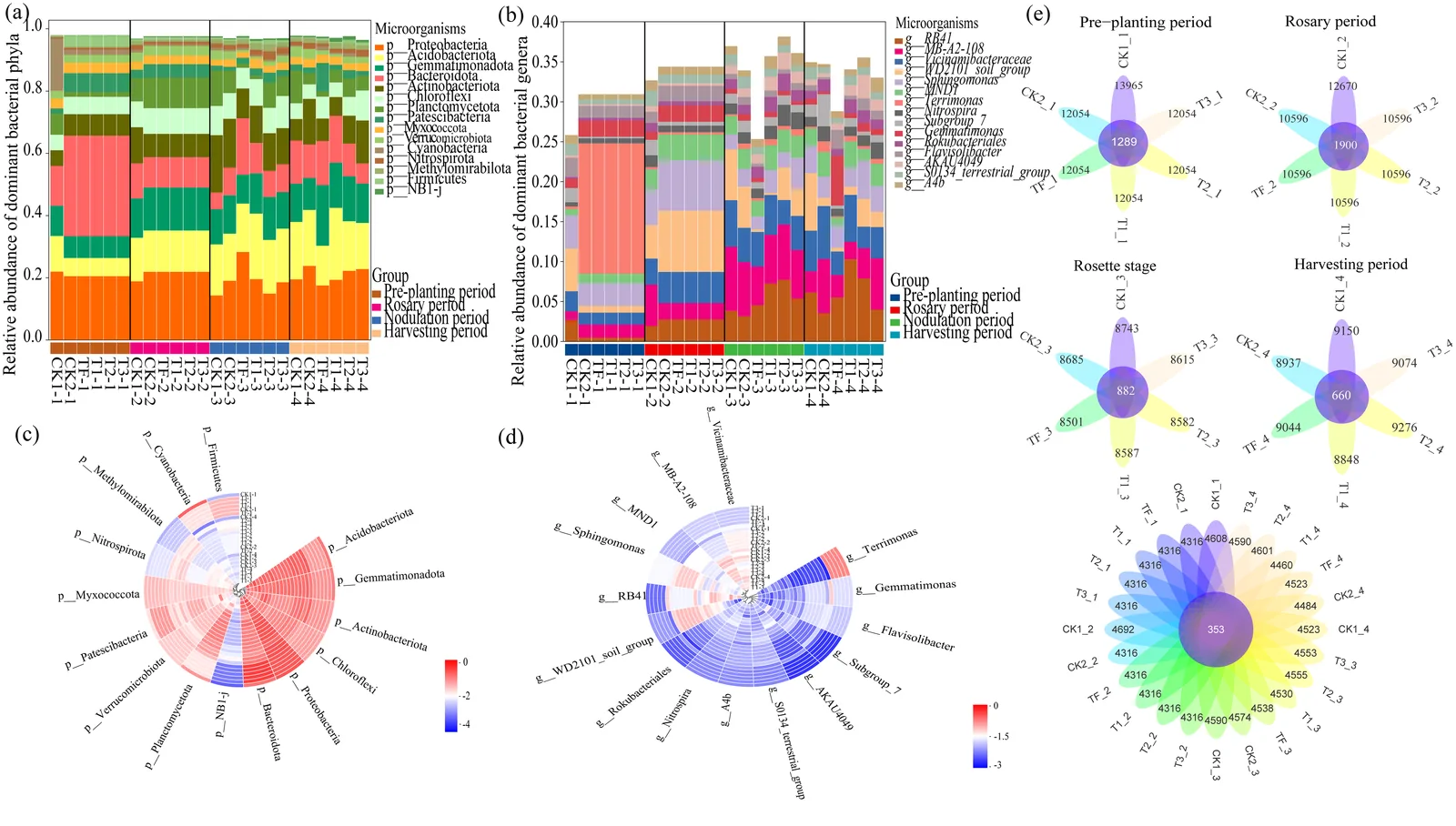
Dynamics of soil microbial community structure, species composition, and OTU distribution under different fertilization regimes during Chinese cabbage growth stages. (**a**,**b**) Relative abundance proportions of dominant bacterial phyla and genera; (**c**,**d**) hierarchically clustered circular heatmaps of major bacterial phyla and genera (log10-transformed); (**e**) petal plot showing shared and unique OTUs of different treatments and universal core OTUs across all growth stages.

Figure 5 illustrates the shifts in soil microbial community abundance and composition under different fertilization regimes. At the phylum level (Figure 5a), *Proteobacteria* dominated across all treatments and represented the most enriched bacterial group in top soil samples. Compared with reduced fertilization and unfertilized controls, supplementary fertilization significantly increased the relative abundance of *Proteobacteria* in the TF. As the second most abundant phylum, *Acidobacteriota* increased markedly at the rosette stage after cabbage planting and reached the highest abundance in T1 among fertilized plots. At harvest, T2 exhibited higher *Acidobacteriota* abundance relative to TF, implying that excessive organic fertilization may suppress *Acidobacteriota* populations. *Acidobacteriota* are vital for soil carbon cycling and plant residue degradation and typically thrive under low pH conditions; their reduction in TF suggested that over application of organic fertilizer could modify soil pH and constrain their growth, consistent with previous observations of pH driven bacterial community alterations under alkaline organic fertilization. The sharp decline of *Bacteroidota* after cabbage planting reflected the impacts of root activity and fertilization on microbial succession. Although TF induced a slight post fertilization rise in *Bacteroidota*, its abundance never recovered to pre planting levels, indicating strong community reshaping by cabbage cultivation. At the genus level (Figure 5b), community composition varied with both fertilization and sampling time. *MB-A2-108*, *Vicinamibacteraceae*, *RB41*, *Sphingomonas*, and *MND1* were predominant genera. *Terrimonas* peaked under basal fertilizer treatments before planting yet decreased sharply after cabbage transplantation, while *WD2101_soil_group* became dominant at the rosette stage, indicating fertilization and crop growth driven microbial succession. TF maintained lower overall microbial abundance throughout the growing season and showed the lowest community abundance at harvest, where *RB41* became dominant, suggesting long term manure input could selectively favour certain bacterial taxa. The enrichment of *MB-A2-108* and *Rokubacteriales* at the heading stage in organic fertilizer treatments (TF, T1, T2) temporally coincided with high ARGs and MGEs abundance, implying that manure stimulated proliferation of specific host taxa may indirectly contribute to short term ARGs enrichment; such enrichment was mitigated under optimized reduced organic fertilization (T2). The circular heatmaps (Figure 5c,d) further depicted phylum and genus level temporal profiles. Copiotrophic taxa such as *Proteobacteria* and *Bacteroidota* were strongly enriched at the heading stage under high organic input (TF), whereas oligotrophic *Acidobacteriota* were suppressed under early stage high nutrient conditions but recovered notably in reduced organic T2 at harvest.

The petal plot based on OTU clustering (Figure 5e) quantified fertilization- and growth-stage-driven differentiation of microbial diversity and core-community overlap. The number of shared OTUs across the six treatments increased from 1289 at the pre-planting stage to 1900 at the rosette stage, suggesting convergent community features stimulated by early-stage crop root effects. Nevertheless, shared OTUs dropped sharply to 882 at the heading stage and 660 at harvest, demonstrating pronounced community divergence among fertilization groups, especially between organic-amended plots (TF, T2) and chemical-fertilizer-dominated T3 as well as unfertilized controls. Across all 24 sample groups covering six treatments and four growth stages, a total of 353 universal core OTUs were identified. These core OTUs constituted the intrinsic microbial backbone of this long-term experimental soil, largely resilient to fertilization interference and phenological variation, and likely underpin fundamental soil carbon- and nitrogen-cycling functions and ecosystem stability. Fertilization-induced microbial successions are known to be time-dependent, shaped by nutrient availability, soil physicochemical properties and microbial adaption to local environments; repeated manure input over planting cycles further remodels soil microbiomes by selecting for particular microbial populations while modifying overall community diversity [30,31,32].

#### 3.5.2. Potential Hosts for ARGs and Microbial Associations

**Figure 6:**
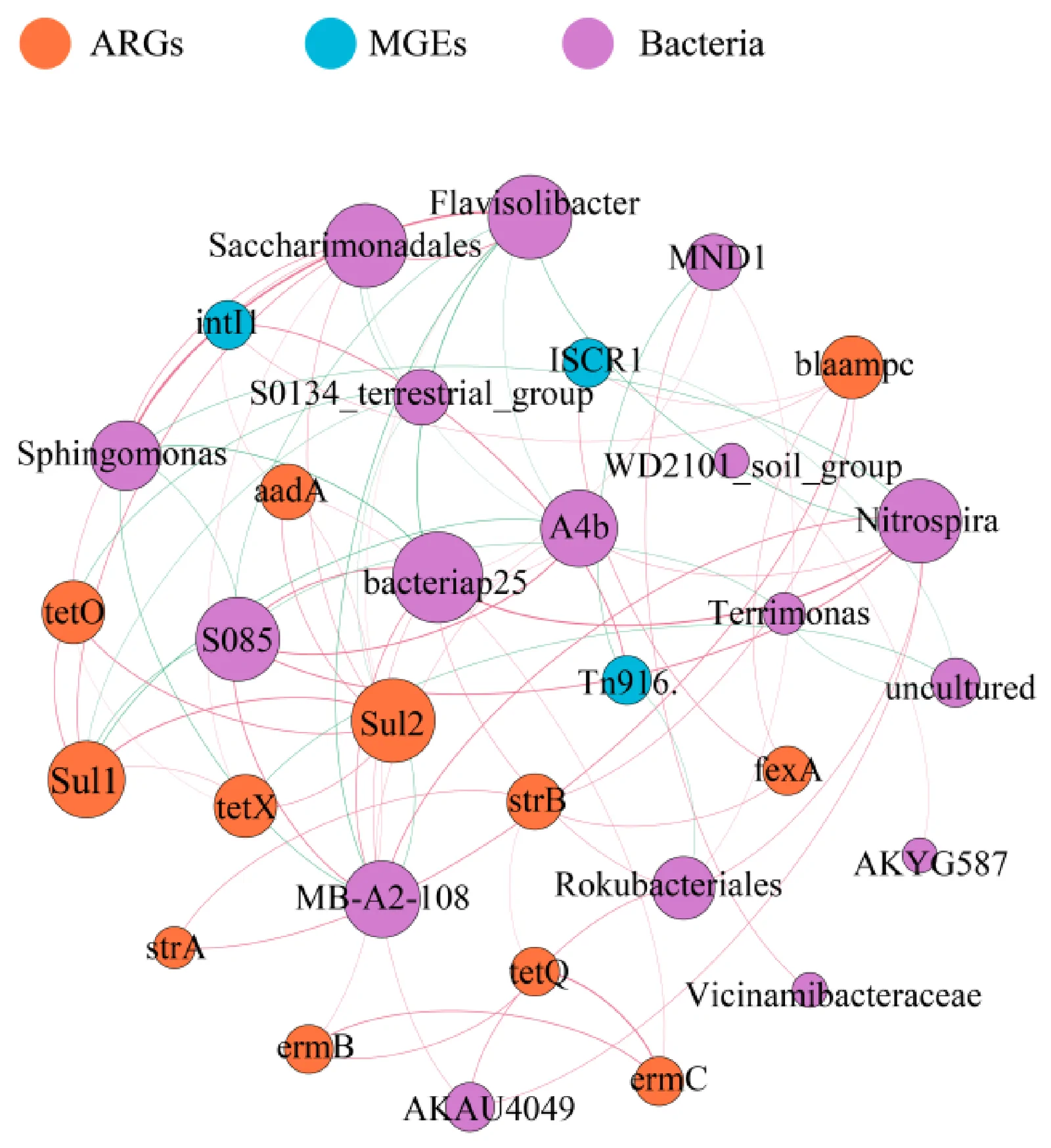
Network analysis of the co-occurrence patterns among ARGs (Antibiotic Resistance Genes), MGEs (Mobile Genetic Elements) and microorganisms in manure applied soil.

Figure 6 presents network analysis of microbial taxa co-occurring with ARGs in manure-amended soils. A total of 17 bacterial taxa were significantly associated (*p* < 0.01) with 12 ARG classes and 3 mobile genetic elements (MGEs), indicating a strong relationship between microbial composition and ARG dissemination.

Among ARGs, *tet*Q, *str*B, and *sul*2 exhibited the highest frequency of co-occurrence with bacterial taxa, suggesting that these resistance genes are widely distributed across multiple microbial hosts. Network analysis revealed a significant positive correlation (*p* < 0.05) between *Saccharimonadales* and *sul*1, while *sul*2 was positively correlated with *MB-A2-108*, *A4b*, *Saccharimonadales*, and *AKAU4049*. The high prevalence of *sul*1 and *sul*2 in soil samples further supports their role as dominant ARGs contributing to resistance variation in fertilized soils.

Several bacterial taxa were associated with multiple ARG types, indicating potential multidrug resistance capabilities. The genera *Rokubacteriales*, *bacteriap_25*, *MB-A2-108*, and *Terrimonas* showed significant positive correlations with *str*B, suggesting that *str*B can be carried by diverse microbial hosts and may contribute to ARG spread in soil environments. Additionally, *Saccharimonadales*, *bacteriap_25*, *MB-A2-108*, *Terrimonas*, *AKAU4049*, *A4b*, and *Rokubacteriales* were linked to multiple ARG categories, particularly *sul*-ARGs, *str*-ARGs, *erm*-ARGs, and *bla*-ARGs, reinforcing their role in ARG transmission.

Among MGEs, *bla*_ampC_ was significantly correlated (*p* < 0.05) with both *ISCR*1 and *intI*1, indicating that *bla*_ampC_ is highly mobile within soil microbial communities. The correlation of *Tn*916. with *A4b*, *Nitrospira*, and *S085*, as well as *intI*1 with *Sphingomonas*, *Saccharimonadales*, and *S0134_terrestrial_group*, suggests that these bacteria may serve as key reservoirs for ARG transfer through horizontal gene transfer mechanisms. The presence of *intI*1, a known integron associated with ARG capture and transfer, indicates an increased risk of genetic exchange among soil bacteria [6,7].

The co-occurrence of ARGs and MGEs in fertilized soils highlights the long-term implications of manure application on soil microbial resistance dynamics. While nutrient-rich manure amendments promote crop productivity, they also select for bacterial taxa that facilitate ARG persistence and mobility, raising concerns about the spread of antimicrobial resistance beyond agricultural ecosystems.

### 3.6. Risk Assessment for the Transmission of ARGs in Soil

The spread of antibiotics and antibiotic resistance genes (ARGs) in agricultural soils poses potential hazards to the ecological environment and human health. To quantify the associated risks, the contamination factor (CF) method was used to evaluate ARGs contamination under different fertilization treatments, while the Risk Quotient (RQ) method was adopted to assess antibiotic pollution levels across various fertilization regimes [34]. These two approaches have been widely applied to determine the contamination of heavy metals, antibiotics and microplastics in soil and aqueous environments [35,36,37]. By calculating the contamination indices of numerous ARGs and antibiotics, this study systematically evaluates the temporal effects of organic and chemical fertilization practices on the environmental persistence and ecological risks of antibiotics and ARGs.

#### 3.6.1. Contamination Patterns Across Growth Stages

Table 4 and Table 5 present contamination indices for 16 ARGs at two critical time points—the heading stage and the harvest stage. The contamination index for *fex*A (CF*_fex_*_A_) was greater than 1 in all six fertilization treatments at the rosette stage, indicating that ARG contamination occurred early in the growing season. However, by the harvest stage, CF*_fex_*_A_ dropped below 1 in all treatments, suggesting that the prolonged cropping period helped reduce *fex*A contamination, likely through natural microbial competition and degradation.

A contrasting pattern was observed for *sul*1, which exhibited a further increase in its contamination index across all fertilization treatments over time. This suggests that sul1 persists longer in soil environments and may be more resistant to degradation or microbial competition.

Among all treatments, TF exhibited the highest overall contamination index, with CFzone reaching 1.96 at the heading stage. This indicates that full fertilization (TF) posed the greatest risk for ARG transmission. T2, which received the second-highest organic fertilizer input, had a CF_zone_ of 1.46, reinforcing that higher manure application levels contribute to ARG persistence in soil.

By the time of harvest, the relative risk associated with most ARGs had declined. Several resistance genes—including *bla*_ampC_, *add*A, *str*B, and *erm*A—showed lower contamination levels, indicating that their abundance was reduced through plant-microbe interactions and natural degradation processes. However, a few ARGs—such as *bla*_TEM-1_ and *tet*O—continued to show increased risk, suggesting that certain resistance genes are more persistent in agricultural soils than others.

At the harvest stage, the CF_zone_ ranking was TF > T2 > T1 > T3 > CK2, which closely mirrored the organic fertilizer application levels. This supports the hypothesis that long-term organic fertilizer use contributes to ARG accumulation, leading to increased ecological risk.

Based on the risk quotient (RQ) evaluation of topsoil and Chinese cabbage (Tables S3 and S4), antibiotic residual risks differed significantly across various fertilization treatments. For topsoil, all groups amended with liquid manure basal fertilizer (CK2, TF, T1, T2, T3) yielded an identical total RQ of 1.53 at the rosette stage (Stage B), with ENR contributing 1.17 and CTC contributing 0.29, collectively indicating high ecological risk (RQ ≥ 1). Notably, the RQ of ENR in T3 reached as high as 5.03 at the heading stage (Stage C), representing an extremely high risk. At the harvesting stage (Stage D), the RQ values of CTC in topsoil of all treatments ranged from 0.15 to 0.64, corresponding to moderate risk.

By contrast, antibiotic residual risks in Chinese cabbage were generally low. The RQ of SMX1 in T1 was 0.31, falling within the moderate-risk range (0.1 ≤ RQ < 1). The RQ of CLA was 0.06 in CK2 and T3, classified as low risk. No obvious antibiotic-related risks were detected in cabbage samples from the remaining treatments.

In summary, ENR and CTC were the primary contributors to soil antibiotic risks, and the rosette stage was the critical high-risk period with exceptionally severe risk observed for T3 at the heading stage. Antibiotic risks in Chinese cabbage were generally controllable, with only SMX1 from T1 requiring extra attention. These results demonstrate divergent migration characteristics of different antibiotics within the soil–crop system. Optimized fertilization strategies should prioritize source reduction in ENR and CTC in liquid manure and rational formulation of pre-harvest safety intervals.

However, while ARG contamination levels declined over time, their potential to enter adjacent water systems through surface runoff and leaching remains a critical concern [38]. Studies have shown that agricultural fields fertilized with manure contribute to the spread of ARGs in surrounding water bodies, with measurable increases in antibiotic-resistant bacteria in groundwater and surface water systems [34]. This cross-system contamination raises significant public health concerns, as ARGs in drinking water can facilitate the horizontal transfer of resistance genes to pathogenic bacteria, increasing the risk of antimicrobial resistance in clinical settings.

These findings highlight the need for policy interventions to mitigate the unintended spread of ARGs beyond soil environments. Stricter guidelines on manure application, including seasonal restrictions to prevent runoff and improved waste treatment protocols, could help limit ARG dissemination into water systems. Additionally, enhanced water filtration and disinfection strategies may be necessary to reduce ARG loads in agricultural runoff before it enters public water supplies. Given the growing concerns surrounding antimicrobial resistance, future agricultural policies must integrate both nutrient management and antibiotic resistance mitigation to ensure food safety and environmental sustainability.

#### 3.6.2. Implications for Sustainable Fertilization Practices

Manure application elevates soil ARG contamination, with excessive inputs under full fertilization (TF) driving peak resistance risks. However, the temporal attenuation of composite contamination indices (CF_zone_) from the heading stage to harvest demonstrates the intrinsic resilience of the soil resistome, despite the persistent elevation of specific determinants such as sul1. Halving organic fertilizer inputs (T2) or adopting a mixed regime (T1) significantly reduces antibiotic residues and ARG dissemination relative to TF, whereas chemical fertilization (T3) minimizes persistent resistance risks. These findings highlight precision organic fertilization as an effective strategy to synchronize crop nutrient demands with resistome mitigation. To prevent cross-ecosystem transmission through runoff, leaching, or crop microbiomes, sustainable agricultural policies must combine optimized manure application thresholds with routine environmental monitoring and improved agricultural wastewater management.

#### 3.6.3. Limitations of the Study

Several methodological limitations should be acknowledged. First, targeted qPCR profiling was restricted to 16 ARGs and 3 MGEs; comprehensive metagenomic sequencing is required to capture the complete resistome and mobilome landscape. Second, co-occurrence network analysis provides indirect evidence of host–ARG linkages rather than functional confirmation of horizontal gene transfer, which necessitates validation through conjugative transfer assays. Finally, off-site migration of resistance determinants via surface runoff and vertical leaching was not directly quantified in this field plot experiment, underscoring the need for future studies to evaluate hydrological transport pathways and long-term ARG persistence in agricultural watersheds.

## 4. Conclusions

The key findings demonstrate that reduced organic fertilization (T2) lowered heavy metal residues and tetracycline antibiotic accumulation by 30–50%, while maintaining cabbage yields comparable to TF. Microbial community analysis revealed that reduced fertilization preserved beneficial phyla like *Acidobacteria* and *Proteobacteria* while inhibiting the proliferation of manure-derived pathogens. Network analysis identified *MB-A2-108*, *Saccharimonadales*, and *Rokubacteriales* as key hosts for multi-drug-resistant ARGs, emphasizing the role of microbial interactions in ARG dissemination. Risk assessment further confirmed that T2 reduced the composite contamination index of ARGs by 25% compared to TF, highlighting its efficacy in curbing long-term ecological risks. However, long-term application of organic fertilizers still poses a cumulative risk, as the amount of ARGs spikes briefly after fertilizer application and then declines over the reproductive period. These results validate that while long-term fertilization is generally environmentally sustainable, optimized organic fertilization strategies can significantly reduce the risk of ARG dissemination while maintaining soil fertility and crop yield.

## Figures and Tables

**Table 1:** Nutrient application rates of different treatments at topdressing stage (kg·hm^−2^).

Treatment	Fertilization Mode	N Input (kg·hm^−2^)	P Input (kg·hm^−2^)
CK1	No fertilizer	-	-
CK2	Basal fertilizer + no additional fertilizer	-	-
TF	Basal fertilizer + full organic and chemical mix	370.00	287.00
T1	Basal fertilizer + half organic and chemical mix	185.00	143.50
T2	Basal fertilizer + half organic fertilizer	185.00	143.50
T3	Basal fertilizer + half chemical fertilizer	185.00	143.50

Note: All treatments were arranged in a randomized block design with three biological replicates to reduce experimental error.

**Table 2:** Types of genes included in the six ARGs.

ARG Class	Target Genes
Streptomycin (*str*-ARGs)	*str*A, *str*B, *aad*A
Macrolide (*erm*-ARGs)	*erm*A, *erm*B, *erm*C
Sulfonamide (*sul*-ARGs)	*sul*1, *sul*2
Tetracycline (*tet*-ARGs)	*tet*O, *tet*Q, *tet*X
β-lactam (*bla*-ARGs)	*bla*_TEM-1_, *bla*_ampC_, *bla*_OXA-1_
Chloramphenicol (*cmr*-ARGs)	*fex*A, *cfr*

**Table 3:** Effects of pig farm slurry substituted on yield of Chinese cabbage.

Treatments	Average Yield/kg·acre^−1^	Contribution Rate of Optimized Fertilization (%)
CK1	1.28 × 10^4^ ± 4.48 × 10^3^ c	-
CK2	2.19 × 10^4^ ± 2.81 × 10^3^ b	28.94
TF	3.16 × 10^4^ ± 2.24 × 10^3^ a	59.55
T1	3.10 × 10^4^ ± 2.73 × 10^3^ a	57.77
T2	3.14 × 10^4^ ± 2.77 × 10^3^ a	58.92
T3	3.12 × 10^4^ ± 7.99 × 10^2^ a	58.35

Note: The data in the table are all repeated 3 times, expressed in the form of average ± standard deviation. If there are the same letters in the same column, the difference is not significant (*p* > 0.05). unfertilized control (CK1), unfertilized baseline control (CK2), traditional full-rate combined manure–chemical fertilization (TF), traditional half-rate combined manure–chemical fertilization (T1), half-dose sole manure fertilizer (T2), and half-dose sole chemical fertilizer only (T3).

**Table 4:** Risk of 16 resistance genes in organic fertilizer-applied soil at heading stage.

	*fex*A	*sul*1	*sul*2	*bla*_TEM-1_	*tet*X	*tet*O	*bla*_amp_c	*bla*_OXA-1_	*tet*Q	*aad*A	*str*A	*str*B	*erm*A	*erm*B	*erm*C	*cfr*	CF_zone_
CK1	1.73	1.44	0.60	0.87	1.76	1.23	0.23	0.32	0.63	0.68	0.29	0.63	0.33	1.07	0.64	0.51	0.81
CK2	1.34	0.37	0.07	1.08	0.13	2.58	1.51	0.93	1.07	1.14	1.52	1.34	0.52	1.23	1.15	1.50	1.09
TF	1.29	4.32	5.65	1.47	3.10	0.44	2.30	0.88	1.18	2.98	0.77	1.53	1.52	1.16	1.24	1.49	1.96
T1	1.75	0.42	0.17	0.74	0.17	0.80	1.54	1.68	0.64	0.79	0.42	0.90	0.62	0.84	0.91	0.67	1.07
T2	1.82	0.46	0.51	0.83	1.08	0.69	0.43	1.87	1.03	1.05	0.72	1.29	0.50	0.92	1.25	0.95	1.46
T3	1.68	0.30	0.10	0.87	0.06	0.61	0.47	1.73	0.85	0.61	0.39	0.73	0.68	0.81	0.79	1.06	1.17

**Table 5:** Risk of 16 resistance genes in soil with organic fertilizer application at harvest time.

	*fex*A	*sul*1	*sul*2	*bla*_TEM-1_	*tet*X	*tet*O	*bla*_amp_c	*bla*_OXA-1_	*tet*Q	*aad*A	*str*A	*str*B	*erm*A	*erm*B	*erm*C	*cfr*	CF_zone_
CK1	0.82	2.21	1.39	0.25	0.31	3.87	0.17	2.59	1.01	0.24	0.33	0.96	0.38	0.67	0.54	0.67	1.03
CK2	0.25	2.04	0.01	1.19	0.06	0.25	0.20	1.41	0.64	0.37	0.44	0.31	0.63	0.54	0.65	0.57	0.60
TF	0.23	5.22	0.33	2.76	0.37	8.64	0.35	1.64	0.89	0.87	0.30	0.71	0.47	0.83	0.97	0.91	1.59
T1	0.22	0.65	0.11	1.73	0.03	3.15	0.16	1.43	0.44	0.55	0.13	0.35	0.41	0.50	0.55	1.07	0.72
T2	0.16	0.93	0.66	2.85	1.29	3.39	0.28	2.29	0.68	0.84	0.50	0.32	0.58	0.57	0.88	0.87	1.07
T3	0.25	0.37	0.03	2.13	0.04	1.42	0.11	1.68	0.64	0.58	0.18	0.30	0.57	1.14	0.62	1.07	0.70

Formulae: CF_i_ = $C_{i}$/$C_{i}-backgound$, where $C_{i}$ indicates absolute gene abundance (copies g^−1^ dry soil) at the heading stage, and $C_{i}-backgound$ represents the baseline abundance prior to fertilization. The integrated index CF_zone_ was calculated as CF_zone_ = $\frac{\Sigma {CF}_{i}}{n}$. Risk-assessment thresholds: CF ≤ 1.0, low or negligible risk; 1.0 < CF ≤ 2.0, moderate risk; CF > 2.0, high risk.

## Data Availability

The original contributions presented in this study are included in the article/Supplementary Materials. Further inquiries can be directed to the corresponding authors.

## Supplementary Materials

The following supporting information can be downloaded at: https://www.mdpi.com/article/10.3390/antibiotics15090821/s1, Table S1 The basic properties of fertilizers and soils; Table S2 The detail information of PCR primer; Table S3 Risk of antibiotics in organic fertilizer-amended soil across the entire crop growth cycle; Table S4 Antibiotic residual risks in harvested Chinese cabbage; Table S5 Two-way ANOVA statistical summary table.
