# Supplementary material for: Optimized Organic Fertilization Mitigates Antibiotic Resistance Gene Dissemination in Manure-Amended Soils: A Field Study on Nutrient–Microbiome–Antibiotic Resistance Gene Nexus During Cabbage Reproductive Cycle
Source: Antibiotics (Basel). 2026 Aug 24;15(9):821. doi: 10.3390/antibiotics15090821 (PMC13603731; doi:10.3390/antibiotics15090821)
Supplement: Supplementary file 1 [file antibiotics-15-00821-s001.zip › Supplementary material.pdf]

Supplementary material

Table S1. The basic properties of fertilizers and soils.

		Soil	Organic fertilizer	Liquid manure
pH		8.68		6.63
Organic matter（$g\cdot kg^{-1}$）		11.89	16.09	
Total concentrations （$mg\cdot kg^{-1}/mg\cdot L^{-1}$）	N	860	1394.4	1410.40
	NH_4^+-N	4.56		528.97
	NO_3^--N	57.50		0.44
	Cr	69.33	33.33	-
	Pb	32.25	17.50	-
	Cd	0.16	-	-
	As	14.59	0.30	-
	Cu	30.14	326.03	0.46
	Zn	89.52	2154.10	8.55
Salinity（%）			0.88	0.61

Table S1 compares the basic properties of soil, organic fertilizer, and liquid manure, focusing on pH, organic matter, nutrient concentrations, heavy metals, and salinity.

The soil has an alkaline pH of 8.68, while the organic fertilizer is slightly acidic at 6.63, which may influence microbial activity and nutrient availability differently. Organic fertilizer also contains higher organic matter (16.09 $g\cdot kg^{-1}$) compared to soil (11.89 $g\cdot kg^{-1}$), indicating its potential to enhance soil quality and support microbial communities. Regarding nutrient concentrations, both organic fertilizer and liquid manure are rich sources of nitrogen, with levels at 1394.4 $mg\cdot kg^{-1}$ and 1410.4 $mg\cdot L^{-1}$, respectively, compared to soil at 860 $mg\cdot kg^{-1}$. Liquid manure, in particular, provides a high level of ammonium nitrogen (528.97 $mg\cdot L^{-1}$), which is readily available for plant uptake, whereas nitrate nitrogen is significantly higher in

soil (57.50 mg·kg^{-1}) than in liquid manure (0.44 mg·L^{-1}).

Table S1 also highlights differences in heavy metal concentrations. Organic fertilizer contains significantly higher levels of copper (326.03 mg·kg^{-1}) and zinc (2154.10 mg·kg^{-1}) compared to soil, which has 30.14 mg·kg^{-1} and 89.52 mg·kg^{-1}, respectively. However, soil has higher levels of chromium (69.33 mg·kg^{-1}) and lead (32.25 mg·kg^{-1}) compared to organic fertilizer. Arsenic levels are considerably lower in organic fertilizer (0.30 mg·kg^{-1}) than in soil (14.59 mg·kg^{-1}), suggesting a reduced risk of contamination from this element when organic fertilizer is used. Additionally, organic fertilizer has a higher salinity (0.88%) compared to liquid manure (0.61%), which may impact soil structure and plant health if overapplied.

Overall, organic fertilizer and liquid manure demonstrate significant potential for nutrient enrichment in soil. However, this study suggests that their use should be carefully managed to mitigate risks associated with heavy metal accumulation and salinity, ensuring sustainable soil health and crop production.

Table S2. Detailed information of PCR primers.

Primer full name		（5’-3’）Primer sequences（5’to 3’）
bla$_{ampC}$	upstream primer	CCTCTTGCTCCACATTTGCT
	downstream primer	ACAACGTTTGCTGTGTGACG
bla$_{OXA}$-1	upstream primer	TATCTACAGCAGCGCCAGTG
	downstream primer	CGCATCAAATGCCATAAGTG
*tet*Q	upstream primer	AGAATCTGCTGTTTGCCAGTG
	downstream primer	CGGAGTGTCAATGATATTGCA
*aad*A	upstream primer	GGTTATCGCCGAAGTATCAACTC
	downstream primer	CACCGTAACCAGCAAATCAATATC
*str*A	upstream primer	GCTAACGCCGAAGAGAACTG
	downstream primer	AGGTGTCCGCAATGAGAACA
*str*B	upstream primer	CGGCTGGCTGGTGATAGAT
	downstream primer	GCGTTGCTCCTCTTCTCCA

*erm*A	upstream primer	TTGAGAAGGGATTTGCGAAAAG
	downstream primer	ATATCCATCTCCACCATTAATAGTAAACC
*erm*B	upstream primer	CCGATACCGTTTACGAAATG
	downstream primer	TACTTTGGCGTGTTTCATTC
*erm*C	upstream primer	GAAATCGGCTCAGGAAAAGG
	downstream primer	TAGCAAACCCGTATTCCACG
cfr	upstream primer	TGTGCTACAGGCAACATTGGAT
	downstream primer	CAAATACTTGACGGTTGGCTAGAG
*fex*A	upstream primer	ATTCTCCCGCAAATAACG
	downstream primer	TCGGCTCAGTAGCATCACG
16S rDNA V3-V4	upstream primer	ACTCCTACGGGAGGCAGCAG
（338-806）	downstream primer	GGACTACHVGGGTWTCTAAT
*intI*1	upstream primer	CTGGATTTCGATCACGGCACG
	downstream primer	ACATGCGTGTAAATCATCGTCG
*ISCR*1	upstream primer	ATGGTTTCATGCGGGTT
	downstream primer	CTGAGGGTGTGAGCGAG
*Tn*916/1545	upstream primer	GACAGTATTAAGCCATCAGAC
	downstream primer	TCTTCCGAACACAATCATCT
*Sul*1	upstream primer	CACCGGAAACATCGCTGCA
	downstream primer	AAGTTCCGCCGCAAGGCT
*Sul*2	upstream primer	CTCCGATGGAGGCCGGTAT
	downstream primer	GGGAATGCCATCTGCCTTGA
bla$_{TEM}$-1	upstream primer	GGTCGCCGCATACACTATTCT
	downstream primer	GGTTAGCTCCTTCGGTCCTC
*tet*X	upstream primer	AGCCTTACCAATGGGTGTAAA
	downstream primer	TTCTTACCTTGGACATCCCG
*tet*O	upstream primer	ACGGARAGTTTATTGTATACC
	downstream primer	TGGCGTATCTATAATGTTGAC

Table S3. Risk of antibiotics in organic fertilizer-amended soil across the entire crop growth cycle.

Groups	Time	SMX1	DMC	CLA	AZI	SMT	SDMD	SMX-2	SDM	OFL	CIP	FLU	ENR	TIL	OTC	TC	CTC	DXC	NOR	ENO	SDZ	SUM
CK1	A	0.00	0.00	0.00	0.00	0.00	0.00	0.00	0.00	0.00	0.00	0.00	0.00	0.00	0.00	0.00	0.04	0.00	0.00	0.00	0.00	0.04
	B	0.00	0.00	0.00	0.00	0.00	0.00	0.00	0.00	0.00	0.00	0.00	0.00	0.00	0.00	0.00	0.00	0.00	0.00	0.00	0.00	0.00
	C	0.00	0.00	0.00	0.00	0.00	0.00	0.00	0.00	0.00	0.00	0.00	0.34	0.00	0.00	0.00	0.02	0.00	0.00	0.00	0.00	0.36
	D	0.00	0.00	0.00	0.00	0.00	0.00	0.00	0.00	0.00	0.00	0.00	0.00	0.00	0.01	0.00	0.02	0.00	0.00	0.00	0.00	0.03
CK2	A	0.00	0.00	0.00	0.00	0.00	0.01	0.00	0.00	0.00	0.00	0.00	0.00	0.00	0.02	0.00	0.19	0.01	0.00	0.00	0.00	0.24
	B	0.00	0.00	0.00	0.00	0.00	0.01	0.00	0.00	0.00	0.00	0.00	1.17	0.00	0.04	0.00	0.29	0.01	0.00	0.00	0.00	1.53
	C	0.00	0.00	0.00	0.00	0.00	0.00	0.00	0.00	0.00	0.00	0.00	0.00	0.00	0.02	0.00	0.05	0.00	0.00	0.00	0.00	0.06
	D	0.00	0.00	0.00	0.00	0.00	0.01	0.00	0.00	0.00	0.00	0.00	0.00	0.00	0.03	0.00	0.26	0.01	0.00	0.00	0.00	0.31
TF	A	0.00	0.00	0.00	0.00	0.00	0.01	0.00	0.00	0.00	0.00	0.00	0.00	0.00	0.02	0.00	0.19	0.01	0.00	0.00	0.00	0.24
	B	0.00	0.00	0.00	0.00	0.00	0.01	0.00	0.00	0.00	0.00	0.00	1.17	0.00	0.04	0.00	0.29	0.01	0.00	0.00	0.00	1.53
	C	0.00	0.00	0.00	0.00	0.00	0.00	0.00	0.00	0.00	0.00	0.00	0.00	0.00	0.00	0.00	0.00	0.00	0.00	0.00	0.00	0.00
	D	0.00	0.00	0.00	0.00	0.00	0.02	0.00	0.00	0.00	0.00	0.00	0.00	0.00	0.11	0.01	0.64	0.03	0.00	0.00	0.00	0.80
T1	A	0.00	0.00	0.00	0.00	0.00	0.01	0.00	0.00	0.00	0.00	0.00	0.00	0.00	0.02	0.00	0.19	0.01	0.00	0.00	0.00	0.24
	B	0.00	0.00	0.00	0.00	0.00	0.01	0.00	0.00	0.00	0.00	0.00	1.17	0.00	0.04	0.00	0.29	0.01	0.00	0.00	0.00	1.53
	C	0.00	0.00	0.00	0.00	0.00	0.01	0.00	0.00	0.00	0.00	0.00	0.00	0.00	0.03	0.00	0.05	0.00	0.00	0.00	0.00	0.09
	D	0.00	0.00	0.00	0.00	0.00	0.01	0.00	0.00	0.00	0.00	0.00	0.00	0.00	0.05	0.00	0.32	0.02	0.00	0.00	0.00	0.41
T2	A	0.00	0.00	0.00	0.00	0.00	0.01	0.00	0.00	0.00	0.00	0.00	0.00	0.00	0.02	0.00	0.19	0.01	0.00	0.00	0.00	0.24
	B	0.00	0.00	0.00	0.00	0.00	0.01	0.00	0.00	0.00	0.00	0.00	1.17	0.00	0.04	0.00	0.29	0.01	0.00	0.00	0.00	1.53
	C	0.06	0.00	0.00	0.00	0.00	0.01	0.00	0.00	0.00	0.00	0.00	0.00	0.00	0.01	0.00	0.08	0.00	0.00	0.00	0.00	0.16
	D	0.00	0.00	0.00	0.00	0.00	0.01	0.00	0.00	0.00	0.00	0.00	0.00	0.00	0.03	0.00	0.18	0.01	0.00	0.00	0.00	0.23
T3	A	0.00	0.00	0.00	0.00	0.00	0.01	0.00	0.00	0.00	0.00	0.00	0.00	0.00	0.02	0.00	0.19	0.01	0.00	0.00	0.00	0.24
	B	0.00	0.00	0.00	0.00	0.00	0.01	0.00	0.00	0.00	0.00	0.00	1.17	0.00	0.04	0.00	0.29	0.01	0.00	0.00	0.00	1.53
	C	0.00	0.00	0.00	0.00	0.00	0.00	0.00	0.00	0.00	0.00	0.00	5.03	0.00	0.00	0.00	0.00	0.00	0.00	0.00	0.00	5.03

D	0.00	0.00	0.00	0.00	0.00	0.00	0.00	0.00	0.00	0.00	0.00	0.00	0.00	0.02	0.00	0.15	0.01	0.00	0.00	0.00	0.18

Note: A, Pre-planting period; B, Rosary period; C, Nodulation period; D, Harvesting period.

Table S4. Antibiotic residual risks in harvested Chinese cabbage.

	CK	CK2	CF	T1	T2	T3
SMX1	0.00	0.00	0.00	0.31	0.00	0.00
DMC	0.00	0.00	0.00	0.00	0.00	0.00
CLA	0.00	0.06	0.00	0.00	0.00	0.06
AZI	0.00	0.00	0.00	0.00	0.00	0.00
SMT	0.00	0.00	0.00	0.00	0.00	0.00
SDMD	0.00	0.00	0.00	0.00	0.00	0.00
SMX-2	0.00	0.00	0.00	0.00	0.00	0.00
SDM	0.00	0.00	0.00	0.00	0.00	0.00
OFL	0.00	0.00	0.00	0.00	0.00	0.00
CIP	0.00	0.00	0.00	0.00	0.00	0.00
FLU	0.00	0.00	0.00	0.00	0.00	0.00
ENR	0.00	0.00	0.00	0.00	0.00	0.00
TIL	0.00	0.00	0.00	0.00	0.00	0.00
OTC	0.00	0.00	0.00	0.00	0.00	0.00
TC	0.00	0.00	0.00	0.00	0.00	0.00
CTC	0.00	0.00	0.00	0.00	0.00	0.00
DXC	0.00	0.00	0.00	0.00	0.00	0.00
NOR	0.00	0.00	0.00	0.00	0.00	0.00
ENO	0.00	0.00	0.00	0.00	0.00	0.00
SDZ	0.00	0.00	0.00	0.00	0.00	0.00

SUM	0.00	0.06	0.00	0.31	0.00	0.06
